# Society Meetings

**Published:** 1909-04

**Authors:** 


### SOCIETY MEETINGS.

---

The Fifth Annual Conference of the Council on Medical Education of the American Medical Association will be held at the Auditorium Hotel, Chicago, Monday, April 5, 1909.

PROGRAM.

10.00 *A. M.—Morning Session.*

Address of the Chairman, Arthur Dean Bevan, Chicago; Report of the Secretary, N. P. Colwell, Chicago; Report of Committee on Medical Curriculum—Anatomy, including Histology and Embryology, Charles R. Bardeen, Professor of Anatomy, University of Wisconsin, College of Medicine, Madison, Wis.; Physiology and Physiologic Chemistry, Elias P. Lyon, Professor of Physiology, St. Louis University, School of Medicine, St. Louis; Pathology and Bacteriology, William T. Councilman, Professor of Pathology, Harvard Medical School, 240 Longwood avenue, Boston; Pharmacology, Toxicology and Therapeutics, Torald Sollmann, Professor of Pharmacology and Materia Medica, Pharmacologic Laboratory, Western Reserve University, Medical Department, Cleveland, O.; Medicine, including Pediatrics and Nervous and Mental Diseases; George Dock, Professor of Medicine, Tulane University of Louisiana, New Orleans; Surgery: General and Special, Charles H. Frazier, Professor of Clinical Surgery, University of Pennsylvania, Department of Medicine, Philadelphia; Obstetrics and Gynecology, Joseph B.

DeLee, Professor of Obstetrics, Northwestern University Medical School, Chicago; Diseases of the Eye, Ear, Nose and Throat, George E. deSchweinitz, Professor of Ophthalmology, University of Pennsylvania, Department of Medicine, Philadelphia; Dermatology and Venereal Diseases, William A. Pusey, Professor of Dermatology and Clinical Dermatology, College of Physicians and Surgeons, Chicago; Hygiene, Medical Jurisprudence and Medical Economics, F. F. Wesbrook, Professor of Pathology and Bacteriology, University of Minnesota, College of Medicine, Minneapolis.

2.00 *P. M.—Afternoon Session.*

Some Results of Higher Standards of Preliminary Education, Richard H. Whitehead, Dean of the Department of Medicine of the University of Virginia, Charlottesville. Discussion.

The Character of the State Medical License Examination, Fleming Carrow, Member of the State Board of Registration in Medicine of Michigan, Detroit. Discussion.

---

The Association of American Medical Colleges held its 19th Annual Meeting in the Academy of Medicine, 17 West 43d street, New York, March 15 and 16, 1909, under the presidency of Professor Eli H. Long, of Buffalo.

The sessions began Monday, March 15, at 10 o'clock A. M., and were devoted to reports of (a) the Committee on Medical Education; (b) Committee on Equipment of Medical Colleges; (c) Committee on State Medical Examining Boards; (d) Committee on Medical Research; (e) Committee on Medical Teaching; (f) Committee on Curriculum.

In the afternoon session, 2 P. M., papers were read as follows: "Higher Entrance Requirements," Hon. Augustus S. Downing, M.A., Ph.D., LL.D., First Assistant Commissioner of Education, Albany, "The Combined Course," Prof. William H. Carpenter, Ph.D., Columbia University; "The Present Status of Medical Education," Dr. Fred C. Zapffe, University of Illinois; "The Five Year Medical Course." Discussion.

The evening session, 8.30 P. M., was held in the Hosack Hall, New York Academy of Medicine, under the auspices of the Academy. Addresses were made by Dr. John A. Wyeth, President of the Academy of Medicine, "The Medical Student in 1867"; Dr. Eli H. Long, University of Buffalo and President of the Association, "Functions of the Medical School"; and Dr. Henry S. Pritchett, LL.D., President of the Carnegie Foundation for the Advancement of Teaching, "Standards of Medical Education." After the meeting a collation was served.

At the morning session Tuesday, March 16, Report of the

Judicial Council, Report of the Secretary-Treasurer and New Business, were made and considered.

---

At the recent annual meeting of the Medical Society of the County of Cattaraugus, a special committee was appointed composed of Dr. A. D. Lake of Gowanda, Dr. Edward Torry of Olean, and Dr. F. C. Beals of Salamanca, to investigate and report as to the advisability of establishing a county biological laboratory, the probable cost of installing equipment and of maintenance, and the most desirable place for locating such an institution.

The election of officers resulted in the choice of Dr. W. W. Jones of Dayton for president; Dr. A. D. Lake of Gowanda, vice-president; Dr. Benjamin Van Campen of Olean, secretary and treasurer. The society voted in favor of a joint meeting of the societies of Cattaraugus, Allegany and McKean counties, to be held at Rock city next summer.

---

The Buffalo Academy of Medicine held meetings during the month of March, 1909, as follows:

*Section on Surgery.*—Tuesday evening, March 2. Program: (1) Description of a Remodeling Operation for Relapsed and Severe Forms of Club Foot, Bernard Bartow; (2) Some Experimental Work on Materials for Plugging Bone Cavities and Sinuses, Prescott LeBreton.

Tuesday evening, March 9, at 8.30 P. M.—Accepted the invitation of The Erie County Pharmaceutical Association to hold a joint meeting of these two bodies and The Erie County Medical Society. This meeting replaced the March meeting of the Section of Medicine. It was held at the rooms of the Society of Natural Sciences, Public Library Building. The Academy was the guest of the Erie County Pharmaceutical Association who presented Dr. William C. Anderson, Dean of the Brooklyn College of Pharmacy. Dr. Anderson read a paper on "The More Extensive Use of U. S. P. and National Formula Preparations to the exclusion of proprietary remedies.

*Section on Pathology.*—Tuesday evening, March 16. Program: (1) Hemolytic Reaction in Sarcoma, H. G. Sloan. Cleveland, O.; (2) Resusitation After Apparent Death From Anesthesia, F. C. Busch, and T. H. McKee.

*Stated Meeting.*—Tuesday evening, March 23. Nominations were made for president, secretary, treasurer and trustee. The section of obstetrics and gynecology provided the following program: Some Interesting Phases in the Life

History of Uterine Myoma, Thomas S. Cullen, Baltimore, M.D. Dr. Cullen is Associate Professor of Gynecology at Johns Hopkins University and Author of Recent Works on Cancer of the Uterus and Fibroid Tumors of the Uterus.

---

At the One Hundred and Third Annual Meeting of the Medical Society of the State of New York recently held at Albany, the following named officers were elected for the current year: president, Charles G. Stockton, Buffalo; vice-presidents, D. C. Moriarta, Saratoga; J. H. Glass, Utica; J. B. Harvie, Troy; secretary, Wisner R. Townsend, New York; treasurer, Alexander Lambert, New York; chairman of the committee on legislation, Frank Van Fleet, New York; scientific work, Leo H. Neuman, Albany; arrangements, W. J. Nellis, Albany; public health, J. L. Heffron, Syracuse.

---

The Rochester Academy of Medicine held meetings during the month of March, 1909, at the Hotel Seneca, as follows.

*Section on General Medicine.*—Wednesday evening, March 3. Program: Visceral Transonance, A. L. Benedict, Buffalo.

Wednesday, March 10. Program: Report of a Case of Unusually Large Sarcoma of both Ovaries, Dr. E. W. Mulligan; Good functional result in compound comminuted fracture of the Olecranon involving the Elbow Joint, Dr. L. W. Rose.

*Obstetrics, Gynecology, and Pediatrics.*—Wednesday, March 24. Program: Psychoses occurring during Pregnancy and the Puerperium, Dr. Eveline P. Ballintine. Discussion was opened by Dr. C. A. Vander Beck.

*Public Health.*—Wednesday, March 17. Program: The New Sanitation, Professor Charles W. Dodge.

